# Surgical Management of a Volvulus of a Wandering Spleen Associated with a Volvulus of the Small Intestine

**DOI:** 10.1155/2022/8696492

**Published:** 2022-04-22

**Authors:** Elisa Maienza, Nathalie Chereau, Fabrice Menegaux

**Affiliations:** Department of General and Endocrine Surgery, Pitié Salpêtrière Hospital, APHP, Sorbonne University, 47-83 Boulevard de l'Hôpital, Paris, France

## Abstract

**Introduction:**

A wandering spleen is a rare anatomical condition characterized by a free-floating splenic tissue that is not located in its normal position in the left upper quadrant. This condition is usually asymptomatic but can also manifest itself with volvulus of the spleen and consequent infarction and necrosis of the parenchyma, requiring an urgent surgical management. Additionally, a wandering spleen can be associated with other contemporaneous anatomical anomalies. *Case Presentation*. We report a case of a 21-year-old woman, admitted to our hospital for intense abdominal pain and vomiting. A CT scan revealed a wandering spleen in the mesogastric area with the spleen torted on its axis, associated with a volvulus of the small intestine. Abdominal exploration revealed a macroscopically normal free-floating spleen attached to an abnormally long vascular pedicle. The management of the wandering spleen was conservative, and a splenopexy was performed.

**Conclusions:**

The torsion of the wandering spleen constitutes an infrequent but life-threatening abdominal emergency. The diagnosis of the wandering spleen is frequently challenging since clinical findings are usually not specific. Imaging such as computed tomography scan plays an important role in the differential diagnosis pathway. Treatment should be planned according to the splenic parenchyma conditions. Splenectomy is indicated when massive infarction and thrombosis of splenic vessels have occurred. When splenic parenchyma is not compromised, it is preferred to perform a conservative surgical technique, such as splenopexy, in order to avoid postsplenectomy complications.

## 1. Introduction

The wandering spleen (WS) is a rare anatomical condition characterized by a free-floating splenic tissue that is not located in its normal position in the left upper quadrant. This is due to the laxity of splenic peritoneal attachments and an elongated vascular pedicle, allowing the spleen to migrate to any part of the abdomen or pelvis. This condition is generally asymptomatic; however, it can also manifest itself with volvulus of the spleen and consequent infarction and necrosis of the parenchyma, requiring urgent surgical management.

Additionally, WS can be associated with other contemporaneous anatomical anomalies, augmenting the complexity of the scenario.

## 2. Case Presentation

We report a case of a patient with volvulus of a WS concomitant with volvulus of the small intestine who reported to the Division of General and Emergency Surgery at Pitié Salpêtrière University Hospital, Paris, in November 2021.

A 21-year-old Caucasian woman presented with a brutal onset of abdominal pain that progressively worsened within the next 24 h. The pain was diffuse, colicky, and nonirradiative. She also complained of nausea, anorexia, and three episodes of vomiting since morning, without frank troubles of transit.

Her past history revealed cardiospondylocarpofacial (CSCF) syndrome in association with cardiopathy, a left megaureter operated on March 2001, congenital deafness, short stature treated with growth hormone during infancy, spine deformities with severe thoracic scoliosis operated on in October 2014, and geleophysic dysplasia.

Her previous surgical abdominal history revealed that she was operated on December 2020 for volvulus of the small intestine due to midgut malrotation, for which Ladd's procedure and appendectomy were performed. No postsurgical complications were noted, except for chronic constipation and occasional mild abdominal distress.

When she arrived at the emergency room, her body temperature was 37.2°C, heart rate was 87 beats/min, and blood pressure was 118/70 mmHg. The Glasgow coma scale score was 15, and the visual analog scale score was 6. The body mass index was calculated as 18.7 kg/m^2^.

On physical examination, spontaneous abdominal pain was present, and on palpation, her abdomen was mildly bloated and sensitive, with no rebound tenderness or palpable masses.

The laboratory results demonstrated an inflammatory syndrome with a white blood cell count of 12.70 × 10^3^/*μ*L and lactate concentration of 1.2 mmol/L.

**Figure 1:**
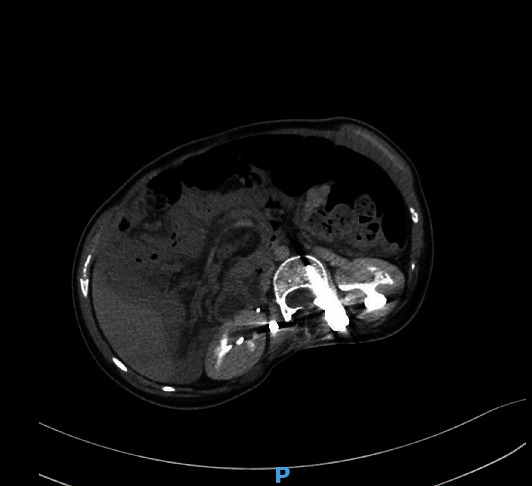
Whirl sign.

**Figure 2:**
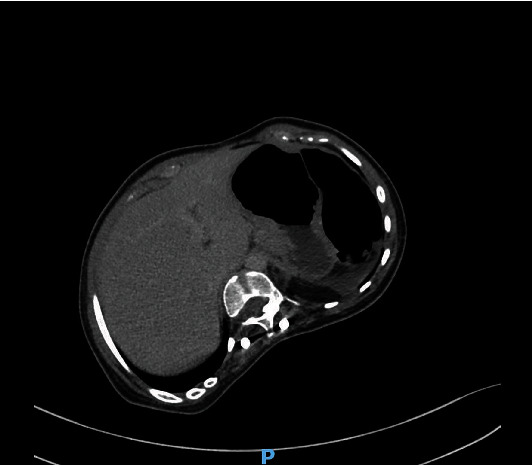
Absence of the spleen in the left hypochondrium.

**Figure 3:**
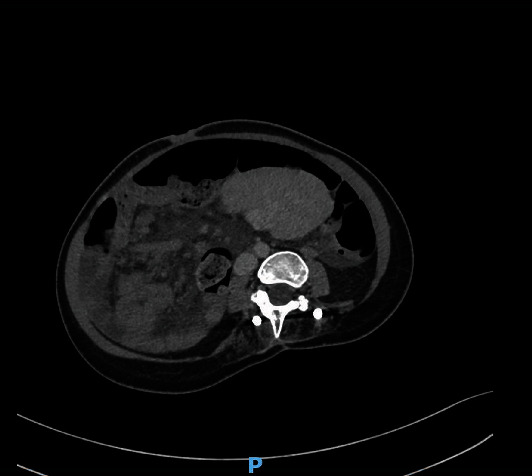
Abnormal position of the spleen in the mesogastric area.

A nasogastric tube was placed, and an abdominal computed tomography (CT) scan with contrast injection was performed because the clinical findings did not point to a definitive diagnosis. CT revealed the absence of distension of the small intestine; however, it demonstrated the rotation of the superior mesenteric vein and artery, with a whirl sign, and a consequent diffuse mesenteric injection (Figure 1). The cecum and terminal ileum were located in the right iliac fossa. The spleen had an abnormal position; it was not located in the left hypochondrium, and was detected in the mesogastric area. The splenic pedicle was elongated and had a whorled appearance in the axial images, with dilatation of the splenic vein. The splenic parenchyma demonstrated homogeneous enhancement on postcontrast imaging; however, no abnormal splenic perfusion was detected (Figures 2 and 3). Minimal free fluid was present in the pelvis and left hypochondria.

Considering the CT scan and clinical findings, we decided to perform an emergency abdominal exploration surgery.

**Figure 4:**
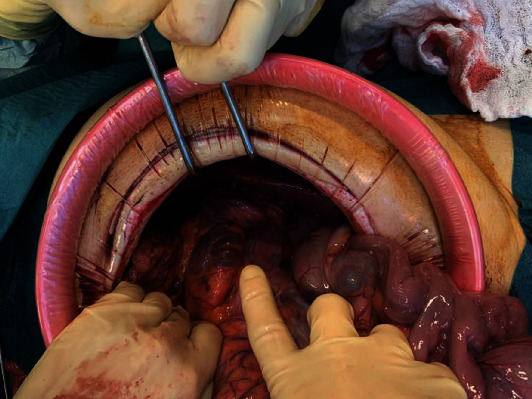
Volvulus of the spleen.

**Figure 5:**
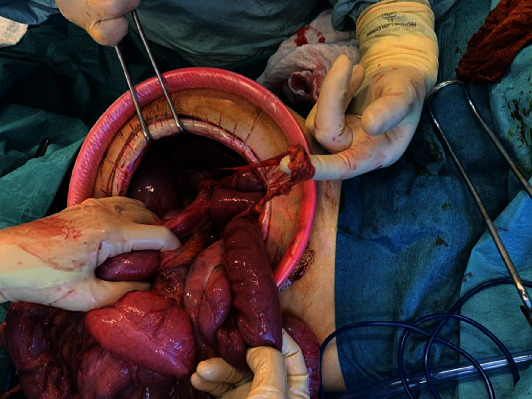
Bridle causing the volvulus of the small intestine.

A median periumbilical laparotomy was performed, with the excision of the old surgical scar. Abdominal exploration revealed a macroscopically normal free-floating spleen attached to an abnormally long vascular pedicle. The vascular pedicle was tortuous along the axis (Figure 4). In addition, volvulus of the small intestine was noted, caused by a long epiploic bridle (Figure 5). There were no signs of intestinal suffering or necrosis. The colon was distended, but without any signs of suffering. The epiploic bridle was dissected; the intestine was completely explored and then placed on the right, while the colon was placed on the left, according to Ladd's procedure.

The spleen was promptly freed. No parenchymal anomalies were observed. The splenic artery was patent and pulsatile. A polyglactin (Vicryl) mesh was then fashioned into a pouch, and the spleen was secured inside it. The pouch was then sutured using polypropylene into the left upper quadrant, replacing the WS in its correct anatomical position. Adequate hemostasis was ensured, and all the layers of the abdominal wall were closed. A course of broad-spectrum antibiotics was administered perioperatively.

**Figure 6:**
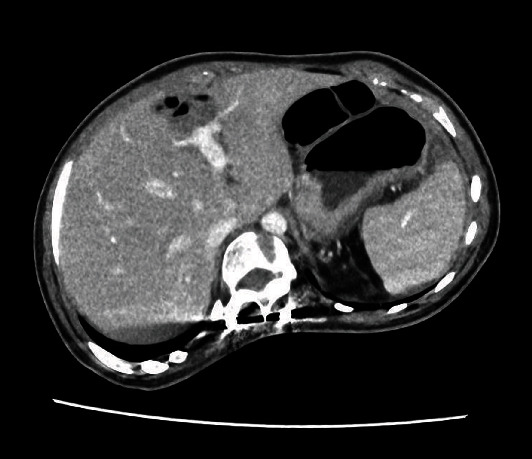
Postoperative CT scan (2 months).

The postoperative course was free of any complications. The nasogastric tube was removed on the second postoperative day, and a clear liquid diet was introduced with good tolerance. Following this, a progressive diet was administered. Transit was restored on the third postoperative day. The patient was discharged on the seventh postoperative day; an abdominal binder was prescribed. At the control visit a month after surgery, the patient was in good health. A CT scan revealed no anomalies with the spleen lying in its natural position, in the left hypochondrium (Figure 6).

## 3. Discussion

WS is a rare anatomical condition characterized by a free-floating splenic tissue that is not located in its normal position, the left upper quadrant. In such cases, the spleen may move to the lower abdomen or pelvic region due to gravity [1]. This is due to the laxity of splenic peritoneal attachments and an elongated vascular pedicle, allowing the spleen to migrate to any part of the abdomen or pelvis [2].

The WS is a distinct condition from the ectopic spleen, which comprises the development of splenic tissue in unusual sites with a normally situated spleen in the left upper quadrant.

In a typical anatomic scenario, the spleen is covered by the visceral peritoneum except for its hilar surface and has very little mobility since it is fixed to the posterior abdominal wall in the splenic fossa by peritoneal attachments. Among these, the most important suspensory ligaments are the gastrosplenic ligament, which contains the short gastric vessels, and the splenorenal ligament, which contains the hilar vessels; the other peritoneal attachments are the splenocolic, splenophrenic, and splenoomental ligaments. The defectiveness of these ligaments due to hyperlaxity, underdevelopment, or absence causes an abnormal positioning of the spleen [3].

The first detailed description of WS in humans is attributed to the Dutch anatomist Johannes Van Horne in 1667. In 1854, the Polish physician Józef Dietl published one of the first case reports of WS in a child [2].

The incidence of this condition is challenging to establish since it is often asymptomatic; however, it is assumed to be approximately 0.15–2% [4–8]. WS has been described in patients of all ages, from 3 months to 82 years [9, 10]; however, it appears to have two peaks of incidence in children aged less than 10 years and childbearing age women [7].

The etiology of WS varies. One cause is the failure of the dorsal mesogastrium to fuse to the posterior abdominal wall during the second month of embryonic development [11], leading to the absence or abnormal development of one or more of the suspensory splenic ligaments [7]. The consequence of this circumstance is an abnormally long splenic pedicle with an increased risk of torsion [1].

WS may also be associated with congenital disorders such as prune-belly syndrome, a multisystem condition characterized by deficient or absent abdominal wall musculature, dilatation of the genitourinary tract, and cryptorchidism, which can interfere with normal gut rotation and fusion of the dorsal mesogastrium [12, 13]. Other anomalies associated with WS are renal agenesis, congenital diaphragmatic hernia, and gastric volvulus [14–16]. Moreover, a familial form has been suggested [17].

There are also acquired anomalies that can be presumably related to WS, such as weakness of the abdominal wall, multiple pregnancies, and hormonal changes. Splenomegaly has also been proposed as a possible etiology; however, in most patients with WS, the spleen demonstrates no anomalies [18]. WS postsleeve gastrectomy has also been described [19].

Furthermore, hyperlaxity of ligaments may also lead to hypermobility of the adjacent viscera, such as the stomach, colon, and pancreas [1, 7, 8, 20]. WS can be associated with dolichomegasigmoid [21] and congenital diaphragmatic hernia [16]. Even though intestinal malrotation and WS can occur independently, these two entities have a common congenital etiology, due to the failure of fusion of the dorsal mesogastrium with the posterior body during fetal development. This condition is responsible for anomalous intraperitoneal visceral attachment [22].

In our case, the patient had CSCF. It is a rare disease caused by a mutation of a gene located in the sixth chromosome, characterized by congenital cardiac defects, growth retardation, vertebral synostosis, deafness, brachydactyly, and typical dysmorphic facial features such as the long face, frontal bossing, and midface hypoplasia [23]. There are no noted correlations in the literature between CSCF and the WS.

As already noted, the majority of patients with WS are asymptomatic, and the condition may be discovered incidentally.

When symptomatic, this condition manifests itself with chronic abdominal pain, due to the abnormally long mesenteries that are prone to intermittent axial twisting [11]. Persistent twisting may also lead to transitory hypoperfusion of the splenic parenchyma, congestion, thrombosis, and ultimately infarction and necrosis of the spleen. Moreover, the mechanical mass effect of WS may cause urinary retention and constipation. The involvement of the adjacent viscera may also be associated with gastric volvulus, acute pancreatitis, and necrosis due to torsion of the pancreatic tail and portal hypertension [15, 24–27].

Imaging findings play a decisive role in the diagnosis of WS and the consequences associated with this condition. Plain abdominal radiography showed the absence of a splenic silhouette in the splenic fossa and the appearance of an abdominal mass, as well as ultrasound. Doppler sonography has a role in underlying the blood flow in the splenic parenchyma and in evaluating the splenic vessels. Finally, a CT scan demonstrates the abnormal position of the spleen and, if twisting is present, vascular congestion signs are associated. It also shows the involvement of the adjacent viscera. In the case of vascular compromise, the splenic parenchyma appears poorly enhanced. It may also show splenomegaly, which is not a specific sign; hyperdense splenic pedicle; pseudocapsular sign; and a whirl appearance of the splenic pedicle when volvulus is present [28–31].

The management of WS may be either nonsurgical or surgical. Conservative nonoperative management is reserved for patients with mild symptoms and no complications that are considered high-risk surgical candidates [32]. This alternative is controversial since it has been noted that 65% of patients with WS undergoing nonoperative treatment have recently developed complications such as splenic torsion requiring surgical treatment [33].

Surgical management of WS has changed over time. Splenectomy was once considered the gold standard treatment of choice. However, the evidence of potential complications in splenectomized patients is in favor of performing spleen-sparing surgical techniques whenever possible. When total splenectomy is performed, an increased risk of potentially lethal infections arises because the protective role of the spleen fails, with increasing morbidity and mortality from infectious complications [34]. The overwhelming postsplenectomy infections are mostly represented by fulminating sepsis, meningitis, or pneumonia, and these conditions are primarily triggered by encapsulated bacteria, such as *Streptococcus pneumoniae*, *Neisseria meningitidis*, and *Haemophilus influenzae* type B in splenectomized and hyposplenic patients. Postsplenectomy and hyposplenic states may also predispose patients to thromboembolic complications [35, 36].

At present, splenectomy is avoided, if possible. Splenopexy is a spleen-preserving surgical procedure that can be achieved when the splenic parenchyma is not compromised. This procedure is aimed at assuring the firmness of the spleen in its natural anatomical site by affixing it and preserving the function of the organ. Various techniques have been described in the literature, and to date, there has been no demonstration of the superiority of any of these procedures concerning long-term results. Both laparoscopic and open approaches appear safe [37].

Ludwick Rydygier performed the first splenopexy in 1895 by fixing the spleen to the peritoneum [2]. Other techniques of splenopexy have been described by Hall et al. in 1903 and Lahey et al. in 1911 [38, 39], using chromic catgut sutures to attach the spleen to the peritoneum.

Stringel et al. fixed the spleen to the natural site via its pedicle [40]. Maxwell-Armstrong et al. used six interrupted 3.0 polydioxanone sutures anchoring the spleen by covering the omentum to the left upper quadrant; a gastropexy was also performed to recreate the gastrosplenic ligament [41]. Another technique involves suturing the spleen by its capsule to the left upper quadrant after the creation of a posterolateral retroperitoneal pouch [42–44]. Caracciolo et al. disconnected the gastrocolic ligament and placed the spleen in its anatomical position, then dislocated the left transverse colon in front of the replaced spleen, and sutured the great curvature of the stomach to the anterior abdominal wall [45]. Schmidt et al. and Allen and Andrews described the technique of splenic snood, consisting of wrapping the derotated spleen into a polyglycolic (Dexon) mesh and anchoring the organ to the diaphragm in the left upper quadrant via the mesh [24, 46, 47]. Splenopexy with a polypropylene pouch has also been reported [11]. Several laparoscopic approaches have been described using polyglactin (Vicryl) or polyglycolic (Dexon) mesh or autologous peritoneal pouch [48–50].

In our case, we chose to perform a splenopexy with a splenic snood, using an absorbable mesh of polyglactin (Vicryl) fashioned into a pouch and anchored to the abdominal wall by polypropylene sutures.

Among the possible postoperative complications of splenic fixation, splenic ischemia, infarction, and recurrent torsion may occur, independent of the chosen technique [51].

## 4. Conclusion

WS is a rare condition that may be associated with severe complications such as infarction and necrosis of the spleen and the involvement of the adjacent viscera, leading to the acute abdomen and requiring emergency surgical management. The diagnosis of WS is frequently challenging. A CT scan plays an important role in the differential diagnosis pathway. Treatment should be planned according to the splenic parenchyma conditions. When massive infarction and thrombosis of splenic vessels have occurred, splenectomy is mandatory. On the other hand, when splenic parenchyma is not compromised, it is preferred to spare the spleen and perform a conservative surgical technique, such as splenopexy. This technique allows avoiding the increased risk of potentially lethal infections and thromboembolic complications occurring after splenectomy.

## Data Availability

The datasets used and/or analyzed during the current study are available from the corresponding author upon reasonable request.

## Abbreviations

WS: Wandering spleen
CSCF: Cardiospondylocarpofacial syndrome
CT: Computed tomography.
